# Analysis and Modeling of the Variations of Root Branching Density Within Individual Plants and Among Species

**DOI:** 10.3389/fpls.2019.01020

**Published:** 2019-08-08

**Authors:** Loïc Pagès

**Affiliations:** INRA, Centre PACA, UR 1115 PSH, Avignon, France

**Keywords:** root branching, branching density, inter-branch distance, model, root system architecture, interspecific diversity, phenotype

## Abstract

Branching density (or the reciprocal: inter-branch distance) is an important trait which contributes to defining the number of roots in individual plants. The environmental and local variations in inter-branch distance have often been stressed, and simulations models have been put forward to take them into account within the dynamics of root system architecture (RSA). However, little is known about the interspecific and intra-plant variations of inter-branch distance. In this paper, we present an analysis which draws on 40 samples of plants belonging to 36 species collected in homogeneous soils, to address how the variations in inter-branch distance are structured within individual plants, and how this structure varies from one species to another. Using measurements of inter-branch distance on various roots of the same species and our knowledge of the branching process, we defined a simple and generic model dedicated to the simulation of the observed variations. This model distinguishes between two sub-processes: i) the longitudinal location of potential branching sites and ii) the effective emergence of lateral roots at these sites. Thus, it represents the variations in distance between the potential sites (with two parameters), and the probability of emergence of a lateral root at each site (one parameter). We show the ability of this model to account for the main variations in inter-branch distances with a limited number of parameters, and we estimated them for the different species. These parameters can be considered as promising traits to characterize—in a comprehensive and simple way—the genetic and environmental variations in the whole branching process at plant level. Based on the results, we make recommendations for carrying out comparable measurements of the branching density in developed plants. Moreover, we suggest the integration of this new model as a module in future RSA simulators, to improve their capacity to account for this important and highly variable characteristic of plant species.

## Introduction

The linear branching density for a given root can be defined as the number of lateral branches per length unit along this root. It can be characterized by the reciprocal variable, the inter-branch distance (IBD), and is often measured on the branching parts of roots, i.e., excluding the distal end where laterals have not emerged yet (Dubrovsky and Forde, 2012).

It is a very important trait at the root system level, because it largely contributes to defining the total number of roots in the whole plant. Since the root system is usually ramified up to several branching orders, typically between 3 and 5, the effect of inter-branch distance on the total number of roots is potentially raised to the power of the number of branching orders. However, note that some species, among the Amaryllidaceae, for example, have roots which are not branched, but they seem to constitute exceptions.

Several authors have quantified IBD (or branching density) in certain species (e.g., banana: Riopel, 1966; Charlton, 1982; Lecompte and Pagès, 2006; maize: Varney et al., 1991; Pagès and Pellerin, 1994; Wu et al., 2016; pea: Hinchee and Rost, 1992; tomato: Barlow and Adam, 1988) and stressed the importance of this morphological trait to characterize the root system architecture of the studied species. Others have compared several species and shown that IBD varies among species and genotypes within species (Mallory et al., 1970; Kong et al., 2014; Bui et al., 2015; Pagès, 2016; Pagès and Kervella, 2018). For example, in a recent paper based on a large range of species, Pagès (2016) observed considerable variations of IBD, with a 10-fold factor between the extreme average values. Within a given species, the variations among genotypes are usually lower, but they were significant for several Solanaceae species (e.g., Bui et al., 2015). Intra-plant variations have received much less attention. Physiologists usually characterize branching density based on the young radicle (Dubrovsky et al., 2006; Lavenus et al., 2013), whereas ecologists propose global average evaluations on mature plants (Kong et al., 2014). Wu et al. (2016) observed intra-plant structured variations, with a dependence of branching density on the diameter of the parent root in field-grown maize plants.

Beyond all these interspecific and intraspecific constitutive variations, the environmental and local plasticity of inter-branch distance have often been stressed, as a key process to cope with heterogeneous soil and locate numerous roots at favorable sites and fewer at unfavorable sites (Drew, 1975; Robinson, 1994; Malamy, 2005; Hodge, 2009; Orman-Ligeza et al., 2018). Several environmental factors were shown to be able to trigger these root responses, such as nutrient (Drew, 1975; Drew and Saker, 1975) or water availability (Orman-Ligeza et al., 2018). The inter-species variations of these plastic responses have not been studied.

Inter-branch distance is a common input parameter in models that simulate the dynamics of the root system architecture, especially in former models (Diggle, 1988; Pagès and Aries, 1988) in which a fixed average value of inter-branch distance was given to each branching order. In other and more recent models (Dunbabin et al., 2013; Henke et al., 2014), inter-branch distance was assumed to be a function which depends on environmental conditions in the vicinity of the root tip. However, the processes included in these latter models were not precisely evaluated against data, because data are lacking concerning both plant response and diversity of root responses. Additional investigations are required to be able to simulate both the variations in IBD within the root system and the plasticity to local conditions.

To inspire this new generation of simulation models, it is interesting to use the outcome of recent physiological works. The processes leading to the construction of branching density have been thoroughly investigated in recent years, usually along the seminal roots of plantlets, mainly in the *Arabidopsis* model (e.g., Dubrovsky et al., 2006; Moreno-Risueno et al., 2010; Dubrovsky et al., 2011; Lavenus et al., 2013). From these studies, it appears that the process of lateral root branching can be valuably divided into several sub processes which occur successively: priming and specification of founder cells in the proximal vicinity of the parent root meristem; initiation of a primordium from these founder cells; development of the primordium, leading to the formation of the apical meristem of the lateral root; emergence of the lateral root from the parent root. Defining these steps allows for a better understanding of the clue signals that operate successively throughout the whole process. For example, a given initial step may define the potential location for branching, whereas a further step may confirm (or not) the branching in that particular place.

Another complementary approach to the topic is to take a macroscopic view of the emergent result of these sequences leading to patterns of inter-branch distances, and to quantify expression variations within the whole root system, taking into account several roots from the same plants and looking at variations among species. In this paper, we present such an analysis based on 40 different samples (36 species) observed in homogeneous soils to investigate how inter-branch distance varies within individual plants and from one species to another. Using this analysis and knowledge of mechanisms, we put forward a new generic and quantitative model to depict and simulate the observed variations.

## Materials and Methods

### Sampling Species

The data used in this paper come from a large data set of plants sampled since spring 2013 either *In Natura* or in pot cultures (**Table 1**). The growing environments were described in several preceding papers (Pagès, 2014; Pagès and Picon-Cochard, 2014; Pagès, 2016; Pagès and Kervella, 2018). Plants observed *In Natura* were obtained in two different French regions that have homogeneous, light, and deep soils. For obtaining these samples, cylindrical soil monoliths (diameter, 25 cm; depth, 30 cm) were extracted around the sampled plants, inserted into a mesh bag, immersed in a large bucket of water, and gently cleaned. Pot-grown plants were cultivated in greenhouses using long PVC tubes (between 50 and 150 cm long, 10 to 15 cm in diameter) filled with either sieved soil or a mixture of sandy soil and sieved compost. Each species was sampled from two to five well-developed plants, usually before flowering. Sampled trees were young, between 2 and 4 years old.

**Table 1:** List of considered samples, with the name of the species, the family, the site of observation (Thouzon and Nozeyrolles represent two different French regions) and the abbreviation used in Figures 1, 4, and 5.

Species	Family	Site	Abbreviation
*Acanthus mollis*	Acanthaceae	Thouzon	AcMoT
*Agrostis capillaris*	Poaceae	Nozeyrolles	AgCaN
*Agrostis vinealis*	Poaceae	Nozeyrolles	AgViN
*Ajuga reptans*	Lamiaceae	Nozeyrolles	AjReN
*Alliaria petiolata*	Brassicaceae	Nozeyrolles	AlPeN
*Amaranthus retroflexus*	Amaranthaceae	Thouzon	AmReT
*Anthoxanthum odoratum*	Poaceae	Pot Clermont-Ferrand	AnOdPC
*Anthoxanthum odoratum*	Poaceae	Nozeyrolles	AnOdN
*Antirrhinum majus*	Plantaginaceae	Thouzon	AnMaT
*Arabidopsis thaliana*	Brassicaceae	Nozeyrolles	ArThN
*Arrhenatherum elatius*	Poaceae	Pot Clermont-Ferrand	ArElPC
*Cirsium vulgare*	Asteraceae	Thouzon	CiVuT
*Dactylis glomerata*	Poaceae	Nozeyrolles	DaGlN
*Geranium molle*	Geraniaceae	Nozeyrolles	GeMoN
*Lactuca sativa*	Asteraceae	Thouzon	LaSaT
*Lolium perenne*	Poaceae	Thouzon	LoPeT
*Mentha suaveolens*	Lamiaceae	Thouzon	MeSuT
*Mercurialis annua*	Euphorbiaceae	Thouzon	MeAnT
*Misopates orontium*	Plantaginaceae	Nozeyrolles	MiOrN
*Panicum capillare*	Poaceae	Thouzon	PaCaT
*Panicum miliaceum*	Poaceae	Thouzon	PaMiT
*Papaver rhoeas*	Papaveraceae	Nozeyrolles	PaRhN
*Plantago lanceolata*	Plantaginaceae	Nozeyrolles	PlLaN
*Poa pratensis*	Poaceae	Pot Clermont-Ferrand	PoPrPC
*Poa trivialis*	Poaceae	Pot Clermont-Ferrand	PoTrPC
*Poa trivialis*	Poaceae	Thouzon	PoTrT
*Prunus domestica*	Rosaceae	Thouzon	PrDoT
*Pseudotsuga menziesii*	Pinaceae	Nozeyrolles	PsMeN
*Rubus ulmifolius*	Rosaceae	Nozeyrolles	RuUlN
*Rubus ulmifolius*	Rosaceae	Thouzon	RuUlT
*Silene vulgaris*	Caryophyllaceae	Thouzon	SiVuT
*Solanum laciniatum*	Solanaceae	Thouzon	SoLaT
*Sorghum halepense*	Poaceae	Thouzon	SoHaT
*Urtica dioica*	Urticaceae	Thouzon	UrDiT
*Verbascum nigrum*	Scrophulariaceae	Nozeyrolles	VeNiN
*Vinca minor*	Apocynaceae	Nozeyrolles	ViMiN
*Vinca minor*	Apocynaceae	Thouzon	ViMiT
*Viola odorata*	Violaceae	Nozeyrolles	ViOdN
*Vulpia myuros*	Poaceae	Thouzon	VuMyT
*Zea Mays*	Poaceae	Pot Avignon	ZeMaPC

From our initial data set, containing more than 200 samples (i.e., a species observed at a given site), we selected only those which had at least 150 branch roots that were measured on parent roots with a sufficient range of diameters to study the effect of this factor. Thus, we kept 40 samples belonging to 36 different species (listed in **Table 1**) that met these conditions for the present study.

### Imaging the Roots

Once the soil had been carefully cleaned, the sampled parts of root systems were spread out in a layer of water contained in a transparent plastic tray. During this operation, lateral roots were carefully spread out on either side of their parent root to facilitate the subsequent measurements. The densest root systems were cut into several pieces to avoid root overlap in the tray. They were then scanned using flatbed scanners equipped with light in the cover (EPSON perfection V700 and V850) at a resolution of 2,400 to 3,200 dots per inch, using the transparent mode. The resolution was adjusted for each species so as to get at least 10 pixels transversally for the finest roots to measure them with sufficient accuracy. Images were stored in jpeg format.

### Measuring the Inter-Branch Distance and Diameter of the Parent Root

Measurements were made on the computer screen by mouse clicking on the displayed images using the measuring tools (i.e., length of straight line and segmented line) provided by the ImageJ software (http://rsbweb.nih.gov/ij/). We measured the diameter of the parent root and the distance along the parent root from each lateral to its proximal closest neighbor (from axis to axis). We quantified branching density based on the inter-branch distance (the reciprocal value), because this variable could be measured for each lateral root. Measurements were done in the young branching zone, where laterals had reached at least 2 mm, to discard the very distal zone where emergence was taking place and where lateral roots might be not visible. Let us note that some lateral roots were broken during washing and imaging, but their trace was clearly visible because of the relative transparency of the parent root cortex.

### Analyzing Data and Modeling

**Figure 1:**
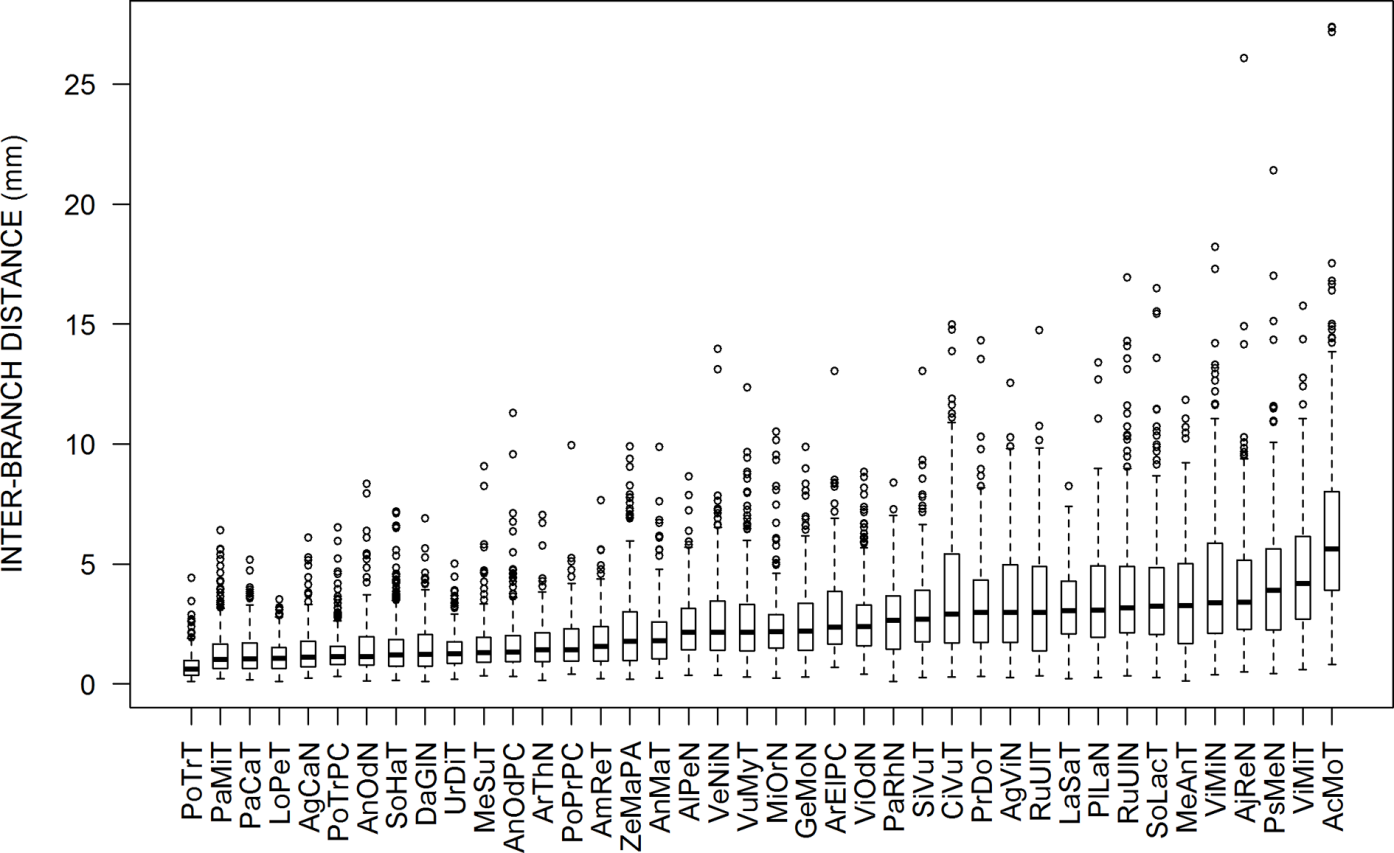
Box plots presenting the distributions of inter-branch distances for each sample. They are sorted from left to right according to the increasing values of the medians. The abbreviations are given in **Table 1**.

**Figure 2:**
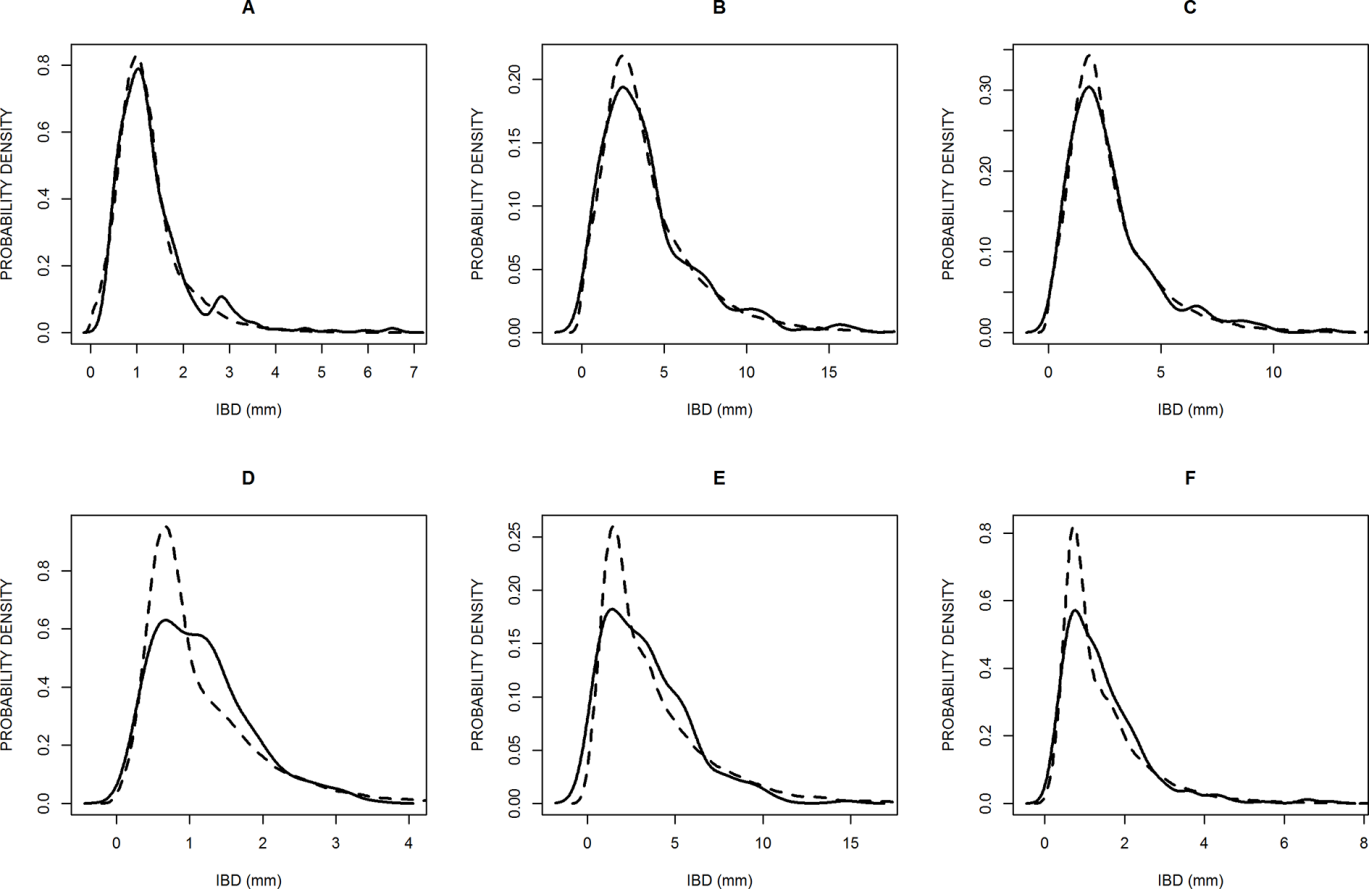
Probability density distributions of six samples to illustrate the best model fittings **(A, B, C)** and the worst **(D, E, F)**, according to the chi square criterion. The solid lines are the observed distributions and the dashed lines are the simulated distributions. The samples are: *Poa trivialis* in Clermont **(A)**, *Solanum laciniatum* in Thouzon **(B)**, *Vulpia myuros* in Thouzon **(C)**, *Lolium perenne* in Thouzon **(D)**, *Rubus ulmifolius* in Thouzon **(E)**, and *Sorghum halepense* in Thouzon **(F)**.

All calculations, plots, and modeling were done using the R software (R Core team, 2013). We made Student *t* tests and analyses of variance using linear models (using the “lm” and “anova” functions), as well as analyses of distributions using several functions of the “moments” package of R (functions: skewness, agostino.test, shapiro.test). We represented the distributions of inter-branch distances graphically using boxplots (**Figure 1**) or using the “density” function that calculates a probability density from the observed or simulated frequencies of inter-branch distances (**Figures 2** and **6**).

Then, we wrote a specific R function (given in **Supplementary Material**) to simulate a model that considers branching as a two-step process. First, potential branching sites are defined along the root, each potential branching site being separated from the previous one (its proximal neighbor) from a distance whose distribution is assumed to be normal. The mean of the distances between potential branching sites is noted *ISD* (inter-site distance), and its coefficient of variation is *CV_D_*. This is the first step that gives a distribution of potential branching sites along the virtual parent root. Second, it is assumed that each potential branching site may actually give rise to an emerged lateral root (i.e., branching success) with a given probability *P*_em_, or it may remain unbranched (branching failure), because one of the following steps could not be accomplished, with a probability of (*1-P_em_*). This is the second step that simulates the positions of the emerged lateral roots along the parent root and allows calculating the distribution of inter-branch distances that can be compared with the observed ones. This simple model does not distinguish between reasons of failure. It could occur during the inception or later development of the primordium or during the emergence as a lateral root. We did not specify the radial angle that defines the orientation of the laterals around the parent root. The proposed model has three parameters: average distance between the potential sites (ISD), coefficient of variation of this distance (CV_D_), and probability (*P*_em_) of success of the whole branching process. These parameters, which quantify the two sub-processes, had to be estimated on the whole, using the observed IBD distributions. The calibrations and tests were done on the whole root population for each species first, and then the model was used to analyze the effects of the diameter of the parent root.

## Results

### Distribution of Inter-Branch Distances

**Figure 1** presents box-plots for the empirical distributions of inter-branch distances (IBD) of all species. Large variations are observed, both within and among species. Within species, the coefficients of variation in IBD varied between 0.5 and 0.8. The median values among species also varied in a large range (9-fold factor) between 0.62 mm (*Poa trivialis* at Thouzon) and 5.63 mm (*Acanthus mollis* at Thouzon). When several samples exist for the same species (*Anthoxanthum odoratum, Poa trivialis, Rubus ulmifolius, Vinca minor*), these samples are rather close to each other.

The IBD distributions were neither normal nor symmetrical. The Shapiro–Wilk normality test rejected the normality hypothesis for all species. The values of skewness and the Agostino test also showed a clear asymmetry for almost all species, with a systematic right-hand tail of the distributions, i.e., an excess of high values above the last quartile, as we can see in **Figure 1** with outliers for high values of IBD. This skewness is reinforced by the fact that we cannot measure negative values of IBD.

When faced with such distributions, it is common to make a logarithmic transformation (e.g., Pagès, 2014; Landl et al., 2018) to obtain a quasi-normal distribution of the response variable [log(IBD)] and to carry out ANOVA on this transformed variable. Using this transformation, we effectively corrected the initial skewness but we obtained an opposite skewness (left tail) for a number of cases (15 of 40 cases according to the Agostino test; normality is accepted in 23 cases according to the Shapiro–Wilk test). As expected, the ANOVA made on this new response variable confirmed a clear species effect (*P* < 0.001).

We also explored the effect of the diameter of the parent root on IBD using correlation tests. Among the 40 samples, the correlation between the diameter of the parent root and IBD was not significant in 12 cases, significant and positive in 5 cases, and significant and negative in 23 cases. Thus, for a majority of species, the laterals tend to be more spaced out on the fine parent roots, but the inverse phenomenon also occurs, although less frequently.

### Fitting and Evaluation of the Branching Model on All Roots

Since the proposed branching model is a numerical and stochastic simulation model, the fitting is not straightforward. To adjust it to the empirical (measured) distributions of IBD, we used a set of intermediate indicators that we constructed first based on simulated distributions and that met three conditions: i) simplicity to estimate these indicators based on the empirical distributions; ii) tight correlations between these indicators and input parameters (ISD, CV_D_, *P*_em_); iii) low correlations between these indicators. For the construction, we made 8,000 simulations of distributions with 60,000 laterals for each of them, combining parameter values distributed within the plausible ranges for each parameter (20 regularly spaced values for each of the three parameters), and we calculated a number of common characteristics for each simulated distribution of IBD: mean, standard deviation, mode, quantiles at probability levels of 0.10, 0.20, 0.30, …, 0.90, 1.0. Based on this preliminary work (not shown), we retained three indicators that fulfilled the three conditions relatively well. They are: the mode of the IBD distribution, which was tightly correlated to the parameter ISD; the ratio: quantile 0.10/mode, tightly correlated to CV_D_; the ratio quantile 0.80/mode, tightly correlated to P_em_. Then, we estimated the three model parameters independently using these three indicators *via* the linear interpolation of the relationships between average parameter values and indicator values that we obtained from preliminary simulations.

We calculated chi square values for each sample to evaluate the quality of the fitting. These values were significant and rejected the hypothesis of identical simulated and empirical distributions in only 3 of the 40 cases (for *Lolium perenne*, *Rubus ulmifolius*, and *Sorghum Halepense* at Thouzon). **Figure 2** shows the three best (**Figures 2A–C**) and three worst (**Figures 2D–F**) fittings obtained. The model distributions are very close to the empirical ones, and the general shape and skewness are very well rendered. A majority of them, but not all, slightly overestimated the probability density of the modal value.

**Figure 3:**
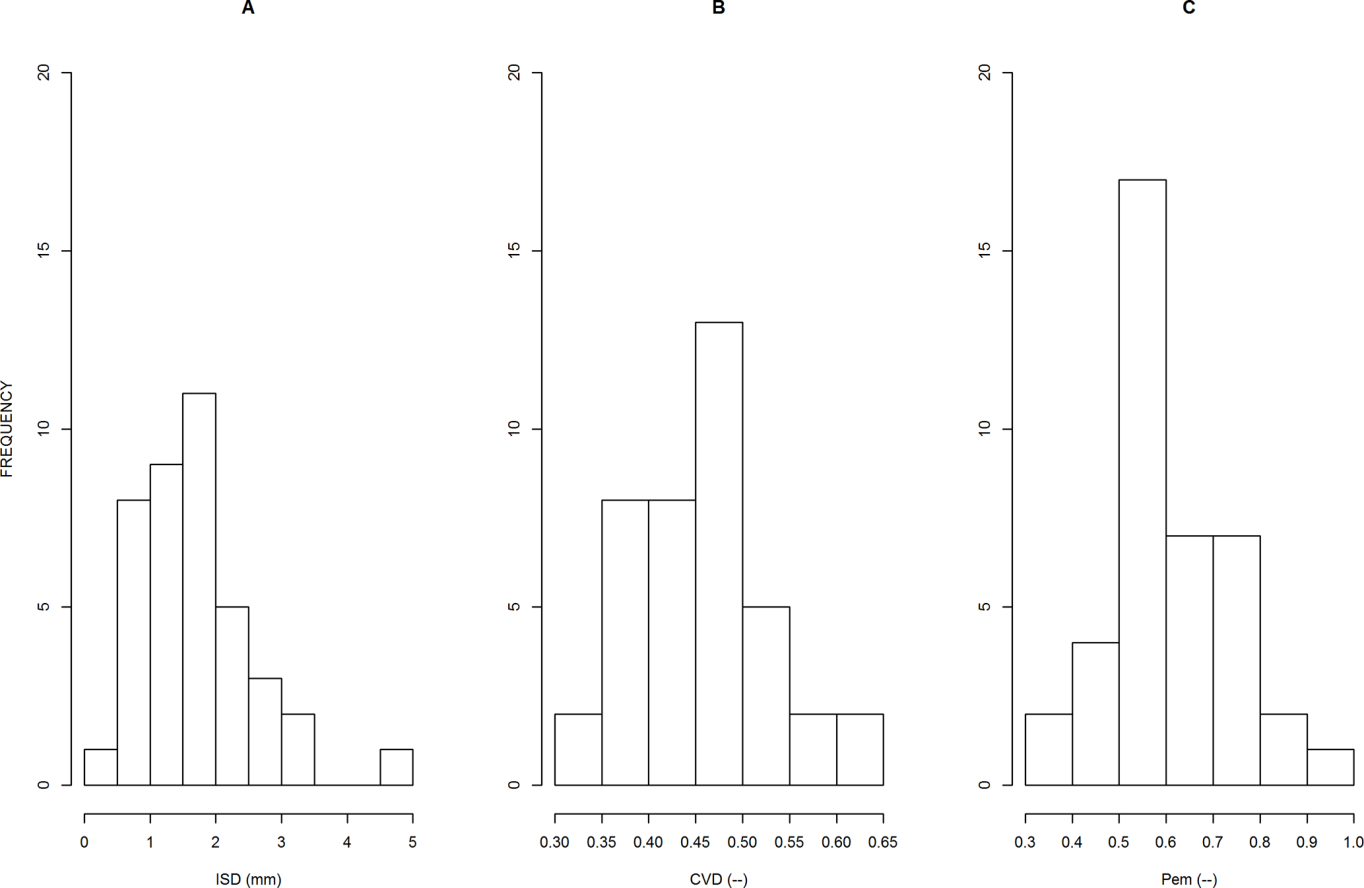
Distributions of the 40 parameter values, as estimated for each sample, for parameter ISD **(A)**, *CV_D_* **(B)** and *P*_em_ **(C)**. The meanings of these parameters are explained in the text.

The distributions of parameters are presented in **Figure 3**. In our population, we obtained very large variations in *ISD*, between 0.40 and 4.6 mm (12-fold factor), confirming the importance of the species effect on this trait. Other parameters were less variable: *CV_D_* varied between 0.28 and 0.69 (2.5-fold factor), and *P*_em_ varied between 0.37 and 0.98 (2.6-fold factor). We also obtained positive and significant correlations between estimates of the three parameters, but the R^2^ values were below 0.3.

### Analysis of the Effect of Parent Root Diameter

For each sample, we divided the population of lateral roots into two sub-populations: those roots originating from parent roots that are finer than the central value of the diameter [0.5*(minimal diameter + maximal diameter)] and those on parent roots with a diameter above this central value. We calibrated the model separately for each sub-population.

**Figure 4:**
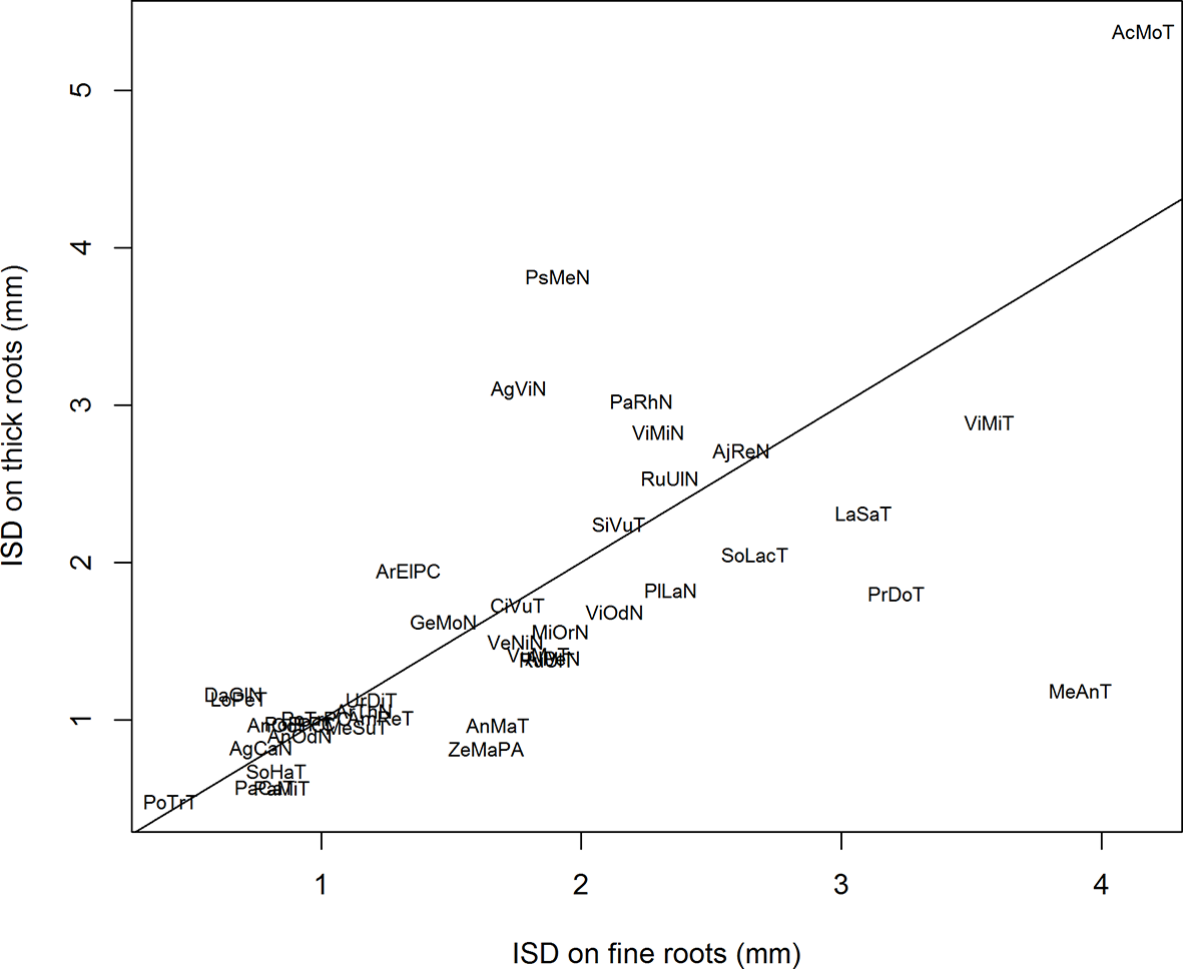
Relationship between the values of *ISD* estimated on thick parent roots versus the values of *ISD* estimated on fine parent roots. The line is the bisecting line. The abbreviations are given in **Table 1**.

**Figure 4** shows the relationship between the *ISD* parameter values as estimated for the two populations. We observed a highly significant correlation between both sets, and we tested that the regression line was not significantly different from the bisecting line (shown in **Figure 4**). A Student *t* test confirmed that there was no global difference between the two sub-populations regarding the *ISD* parameter. For the two other parameters (*CV_D_* and *P_em_*) on the contrary, we did not observe correlations between the two estimates. Again, the Student *t* test did not reveal differences between the two sub-populations. However, the values of *P_em_* tended to be slightly higher for thick roots in a number of species, confirming a tendency for these species to have a heavier right tail in their IBD distribution for the finer roots.

**Figure 5:**
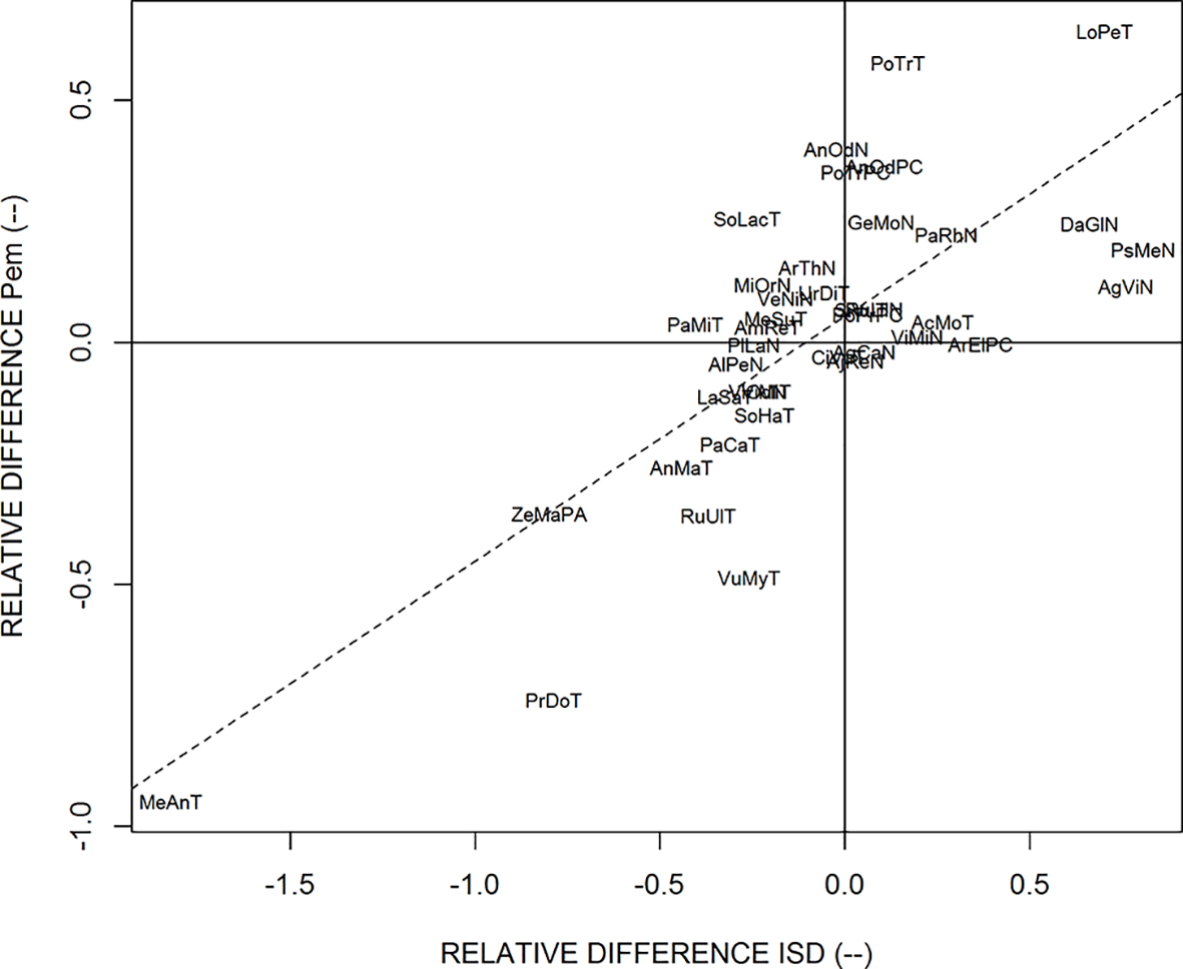
Relationship for the relative difference between the estimates of *ISD* and *P*_em_, when estimated on thick parent roots and fine parent roots separately. The dotted line is the regression line. The abbreviations are given in **Table 1**.

**Figure 6:**
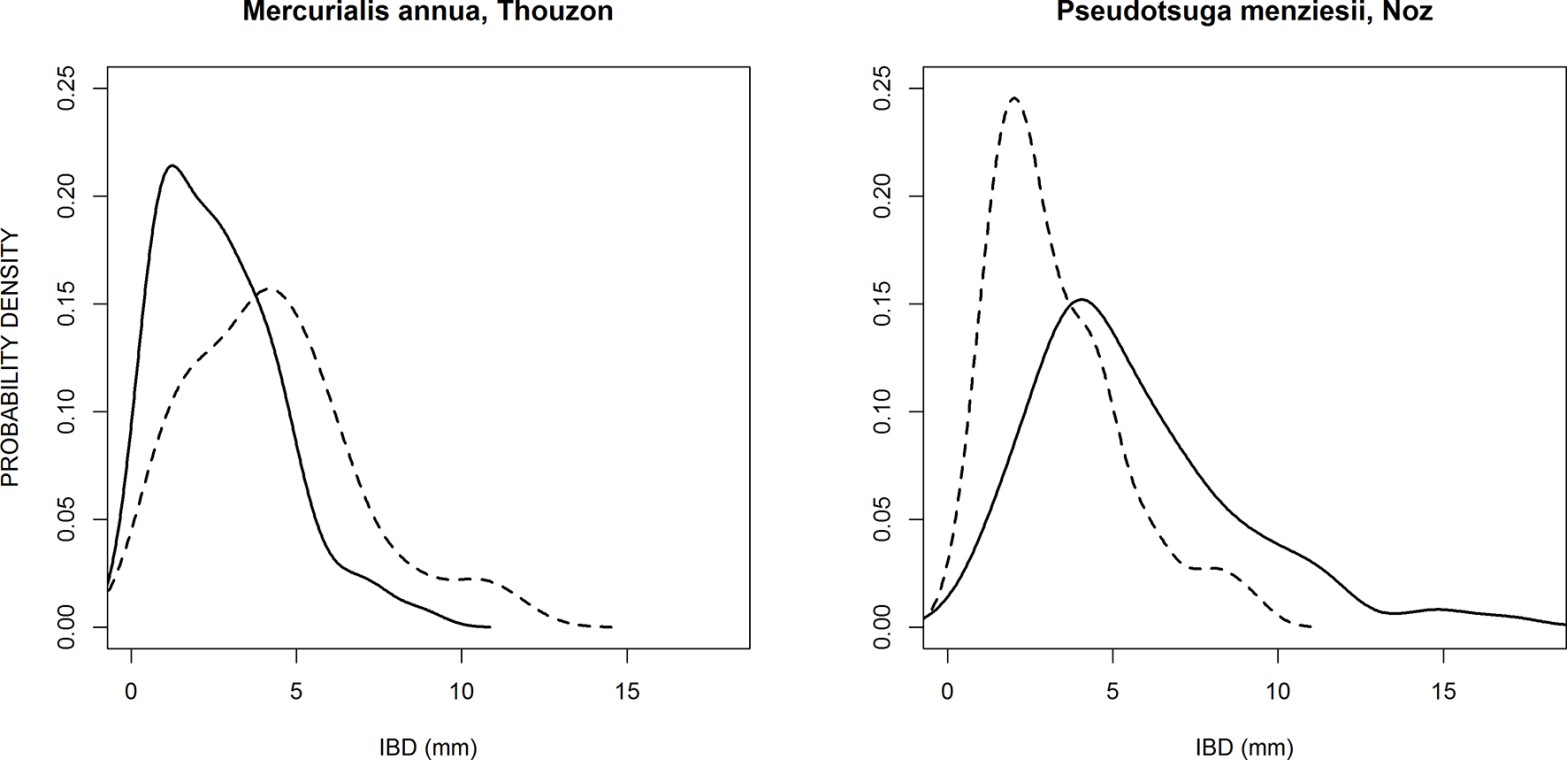
Probability density distributions of two species that exhibited large and opposite differences between the branching patterns of thick parent roots (solid line) and fine parent roots (dashed line).

We also observed that the relative differences of estimated *ISD* were correlated to the relative differences of *P*_em_, as shown in **Figure 5**. This highly significant correlation means that when the potential branching sites are more spaced out in one of the two sub-populations, this is compensated by a higher probability of emergence of laterals on these potential branching sites, and vice-versa. Most species are close to the center of this graph, which shows that they have similar branching parameters for thick and fine roots, but several species exhibited the compensation between the spacing of branching sites and the probability of emergence. This was the case for *Merculialis annua* observed in Thouzon and *Pseudotsuga menziesii* observed in Nozeyrolles, whose IBD distributions are represented in **Figure 6**. These species showed extreme behaviors regarding distributions: for *Mercurialis annua*, the lateral roots on fine roots were more spaced out, whereas *Pseudotsuga menziesii* showed higher densities on its fine roots.

## Discussion

### Main Characteristics of the Distributions of IBD

In this study, we confirm that IBD exhibits large variations in each of the 40 species samples, both within the species and between species, as reported for example by Mallory et al., (1970); Kong et al., (2014); Bui et al., (2015); Landl et al., (2018), or Pagès and Kervella (2018). In most works, however, intra-species variations were not studied and were dismissed as noise. These variations are often smoothed over using other measure variables, such as branching density, evaluated by various counting protocols relying on root segments, whole roots, or even whole root systems. The problem is that such estimates of branching density are hardly comparable from one study to another because the obtained values are more or less buffered, and they depend on the developmental stage of the root or root system. The point was thoroughly discussed by Dubrovsky and Forde (2012). Following their main recommendations, we measured this IBD trait in young, although fully branched, parts of individual roots, because such measurements are more suitable to reflect the underlying developmental process of acropetal branching, even though highly variable measurements are obtained when following this method. The point is even more important when the objective of the study is to link the branching process to local environmental variables, such as water and nutrient availability. In our study, we did not consider the radial pattern of emergence that is usually influenced by the internal vascular structure. It would have been difficult because the number of vascular poles varies from one root to the other, and it may even vary along the roots (personal observations).

Despite the large intra-plant variations of IBD, the inter-species variability was so high (nine-fold factor in our study) that the species effect was significant. Because of the large intra-plant and intra-root variations, testing a species or genotype effect requires a sufficient number of measurements for each one of them as well as measurements made in very comparable situations for all. In this study, we had at least 150 measurements per sample combined with 40 different samples. The species effect was not specifically the subject of the present study, but it was demonstrated earlier by Pagès and Kervella (2018) using a dedicated sampling design for the purpose. Our large sampling numbers were also justified because in addition, we intended to evaluate the intra-sample variations associated to the diameter of the parent roots.

### Interest of Modeling

This model is the first attempt to simultaneously represent several aspects of the longitudinal branch distribution that have been observed by a number of authors, but on a very limited number of species, using either detailed morphological descriptions (e.g., Riopel, 1966; Riopel, 1969; Charlton, 1975; Charlton, 1982; Charlton, 1983; Varney et al., 1991; Hinchee and Rost, 1992; Newson et al., 1993; Charlton, 1996; Draye, 2002; Landl et al., 2018) or physiologically oriented studies (Dubrovsky et al., 2006; Moreno-Risueno et al., 2010; De Smet, 2012). The main aspects that were reported by several of these authors are that regular longitudinal spacing between laterals tends to be maintained along the parent roots, and that the distributions of IBD are usually skewed with a tail toward the large values (Pagès, 2014; Landl et al., 2018). Using our simple model with three input parameters, these aspects, as well as the general shape of the IBD distributions, can be rendered and quantified in a comprehensive and parsimonious way. This study, which relies on a large number of species, is a way of validating the model and asserting its robustness. Thus, the modeling approach opens the possibility of linking highly focused studies that investigate underlying mechanisms with global and macroscopic observations that aim at simply characterizing the emergent branching pattern, because of its important functional significance. This link helps quantify simply and separately several sub-processes: the creation of the archetypal distance (model parameter *ISD*), its variations (model parameter *CV_D_*), and the probability of accomplishing the whole branching process until lateral emergence takes place on each branching site (summarized by the *P_em_* parameter). These different sub-processes are worth separating because they occur successively, involve various molecular mechanisms, and respond to different environmental stimuli (Dubrovsky et al., 2006; De Smet, 2012).

This model can be integrated as a component (or module) in larger models of the root system architecture (Dunbabin et al., 2013). Branching density is a central process in such models, having the prime role in defining the number of roots and eventually the total root length of the whole root system. Several models of the root system architecture use an average IBD input parameter calibrated for each category of roots, defined by their branching order or other typology. Our results suggest that there are differences between roots regarding their branching distribution, but they are not independent, since the *ISD* parameter estimates were significantly correlated for fine and thick roots. Therefore, we suggest using a common *ISD* parameter for all roots and adapting the other important parameter (*P_em_*) to the parent root diameter and possibly to the root position in the surrounding soil. This method would lead to a reduced number of parameters with a more clear and biological meaning.

### Use in a Phenotyping Perspective

The parameters of the present model may also be used as interesting traits for root phenotyping approaches. Among the three parameters, we can guess that *ISD* is rather genetically controlled, whereas *P*_em_ may be more sensitive to local conditions and would reflect plasticity. These speculations based on the present results are worth verifying using suitable experimental designs that cross genotypes and environmental conditions. We have seen that *ISD* can easily be estimated based on the mode of the empirical distribution of IBD, which represents the most frequent inter-branch distance. This value seems relatively easy to obtain, and could be estimated on a reduced number of lateral roots, several tens for instance. The probability parameter *P_em_* has a very different impact on distribution. Its value modifies the right tail of the distribution, so that it can be conveniently estimated *via* the high-probability quantiles of the distribution. We used the 0.8 level in the present study. According to our model, a Gaussian distribution is obtained as the limit distribution when *P_em_* = 1, i.e., when all branching sites give rise to an emerged lateral root. The importance of the right tail increases with decreasing values of *P_em_*. Therefore, *ISD* would summarize a genetic potential for the plant regarding branching density, and *P_em_* would summarize the accomplishment of this branching density in the given environmental conditions. The impact of the third parameter (*CV_D_*) on the global distribution is lower, and its estimation is more difficult. Thus, its interest from a phenotyping perspective is much lower.

### Exploration of Intra-Plant Variations

Thanks to our original data set which relies on many different species and well-developed and highly branched root systems, we could study the inter-branch distance on main roots as well as lateral roots, and chose to study the effects of the diameter of the parent root. We showed that in most species, the *ISD* values were not impacted by the parent root diameter. Thus, for all these species, the fineness of roots did not modify this archetypal distance. On the other hand, the diameter of the parent root had a higher -although unpredictable- impact on the probability of accomplishing the branching process (*P_em_* parameter). For a majority of species, this probability decreases with decreasing diameters, but it is not a generally observed phenomenon. We also observed opposite cases, where it seems that failure is more probable for thick roots than fine roots.

Since the differences observed for the values of ISD and Pem in the sub-populations of parent roots (thick versus fine) were not independent, we can suspect that it might reflect different plant strategies regarding the positioning of their laterals. Some species had higher densities on their main thick roots, whereas others favored higher densities on their fine roots. Both strategies can make sense and participate in the development of a diversity of branching patterns, in coordination with other developmental processes, as described by Pagès (2014) and Pagès (2016).

It would be very interesting to use this diversity to further explore the molecular mechanisms explaining that laterals have a higher chance to emerge on thick or fine roots within the same genotype.

## Conclusion

Therefore, studying simultaneously intra-plant and inter-species variations and using well-developed root systems is an interesting means of raising new questions regarding both the diversity in the foraging strategies of plants and regarding the physiological mechanisms underlying this diversity. The construction and use of such a simple and generic model is a prime way of allowing the quantitative exploration of this diversity.

## Data Availability

The datasets generated for this study are available on request to the corresponding author.

## Author Contributions

LP made the conception, data acquisition and analysis, and wrote the paper.

## Conflict of Interest Statement

The author declares that the research was conducted in the absence of any commercial or financial relationships that could be construed as a potential conflict of interest.
