# Supplementary material for: Analysis and Modeling of the Variations of Root Branching Density Within Individual Plants and Among Species
Source: Front Plant Sci. 2019 Aug 8;10:1020. doi: 10.3389/fpls.2019.01020 (PMC6694179; doi:10.3389/fpls.2019.01020)
Supplement: Supplementary file 1 [file DataSheet_1.pdf]

Supplementary material

Code of the R function that simulates the proposed model, using the 3 input parameters

```
simulIBD <- function(mean.isd,cvar.isd,pemerge,nb.prim=1000) {
  # Function that simulates the distribution of IBD
  # taking into account the 3 model parameters:
  # - mean distance between the sites (mean.isd),
  # - coefficient of variation of this distance (cvar.isd),
  # - probability for full developmental success and emergence (pemerge)
  # The number of initiated primordia can be specified by nb.prim

  interSiteDistance <- rnorm(n=nb.prim,mean=mean.isd,sd=mean.isd*cvar.isd) # distances between
the sites of primordia
  positionSites <- cumsum(interSiteDistance)  # longitudinal positions of sites along the parent root
  latEmerge <- runif(n=nb.prim,min=0.0,max=1.0) <  pemerge  # boolean sequence specifying
emergence or not
  positionLat <- positionSites[latEmerge] # longitudinal positions of emerged laterals
  IBD <- diff(sort(positionLat))  # inter-branch distances
  return(IBD)
}
```
